# Assessment of Quality Indices in Japanese Quince (*Chaenomeles japonica* L.) Juice and Concentrate: Evaluating the Impact of Hydrolytic Enzymes and Clarifiers

**DOI:** 10.17113/ftb.63.04.25.8413

**Published:** 2025-12-26

**Authors:** Dalija Segliņa, Inta Krasnova, Vitalijs Radenkovs, Karina Juhņeviča-Radenkova, Ingmārs Cinkmanis, Linards Kļaviņš, Danija Lazdiņa

**Affiliations:** 1 Institute of Horticulture, Graudu Street 1, 3701 Ceriņi, Krimūnu District, Dobeles Municipality, Latvia; 2 Latvia University of Life Sciences and Technologies, 2 Lielā Street, 3001 Jelgava, Latvia; 3 University of Latvia, Faculty of Science and Technology, Department of Environmental Science, Jelgavas Street 1, 1004 Riga, Latvia

**Keywords:** enzymatic treatment, cloudy juice, titratable acidity, pectin, chemical compounds, antiradical activity

## Abstract

**Research background:**

Japanese quince (*Chaenomeles japonica* L.) is known for its relatively high contents of bioactive compounds, including phenolics, vitamin C, organic acids, dietary fibre and pectins. Its acidic nature makes Japanese quince juice a potential alternative to lemon juice, offering preservative and acidifying properties in various products.

**Experimental approach:**

The aim of this study is to evaluate the effects of different hydrolytic enzymes (EnartisZym 1000 S, EnartisZym RS and EnartisZym EZ Filter) and the clarifying agent bentonite Neoclar AF (a blend of bentonite and activated carbon) on the quality indicators of Japanese quince juice and its concentrate. Juice was extracted from frozen fruits then clarified, filtered and concentrated by open water evaporation at 60 °C to achieve approx. 50 % Brix.

**Results and conclusions:**

The results show that hydrolytic enzymes and clarifiers effectively reduced pectin content in the juice and concentrate by 68.5 and 57.0 %, respectively, and thus significantly improved their clarity, namely by 85.9 and 64.9 %. The highest clarity was observed in samples treated with EnartisZym 1000 S and bentonite (0.028) compared to the control. Enzymatic treatment had a minimal impact on phenolic content, with the enzyme-treated juice containing an average of 318.3 mg/100 mL and the concentrate 3.12 g/100 g, compared to 326.2 mg/100 mL and 3.34 g/100 g in untreated samples, respectively. The retention of vitamin C was high, with enzyme-treated juice containing 68.3–69.4 mg/100 mL and the concentrate containing 231.4–236.9 mg/100 g, compared to 72.8 mg/100 mL and 244.9 mg/100 g in untreated samples, indicating that enzymatic treatment and mild processing effectively preserved ascorbic acid content. The DPPH˙ radical scavenging activity was significantly higher in the enzyme-treated juice, although it decreased during juice concentration. In both juice and concentrate, FRAP values were lower in the enzyme-treated samples than in the controls. Based on generalized scores obtained from analytical hierarchy process analysis, the enzyme EnartisZym EZ Filter, which had both cellulolytic and pectolytic activities, was found to be the most efficient, ensuring the quality characteristics of Japanese quince juice concentrate nearly equivalent to those of commercially available lemon juice concentrate.

**Novelty and scientific contribution:**

This study provides valuable insights into the potential of Japanese quince juice concentrate as a natural acidifying alternative to lemon juice, addressing a gap in existing research. The obtained data show that the concentrate not only offers preservative and acidifying properties, but also retains significant nutritional benefits, making it a promising ingredient for food applications. As an innovative niche product, Japanese quince juice concentrate could enhance food preservation and quality naturally. Future research should further investigate its applications in various food formulations to maximize its functional and commercial potential.

## INTRODUCTION

The *Chaenomeles* genus is globally recognized as an ornamental plant; however, its cultivation as a fruit crop for processing has a more recent history. The cultivation of *Chaenomeles japonica* L. (Japanese quince) for fruit production in Latvia began in the last century, with the first large plantations established in the 1970s (*1*). Over the past two decades, Japanese quince cultivation has gained popularity in northern European countries, particularly in the Baltic Sea region, with Latvia’s cultivation area currently reaching 752 hectares (*2*).

The fruit of the Japanese quince has a distinct sour taste and a low pH, making it unsuitable for fresh consumption. However, it is widely processed into various products, including juice, puree, syrup, candies, jams and marmalades (*3*). Additionally, less common products such as alcoholic beverages (wine, liqueurs), fruit chips, freeze-dried fruit pieces, and powder have also been developed (*3*). Japanese quince fruit is rich in bioactive compounds such as polyphenols, vitamin C, organic acids, dietary fibre and pectins (*4*, *5*). It is particularly high in polysaccharides, including cellulose, hemicellulose and pectin – key components of dietary fibre. The pulp contains a significant amount of pectin, with soluble pectins predominantly present in the juice (*6*).

Similar to lemon juice, Japanese quince juice can serve as a natural acidifier (*7*) while also providing additional antioxidant properties to food products (*8*). It is either comparable to or richer in bioactive compounds than lemon juice. For instance, the ascorbic acid concentration in Japanese quince juice ranges from 45–78 mg/100 mL, compared to 23–40 mg/100 mL in lemon juice (*8*, *9*). Although the total acid content is higher in lemon juice (5.2–6.9 %), Japanese quince juice contains a substantial amount of organic acids (2.6–5.6 %) (*10*). Furthermore, the concentration of polyphenolic compounds in Japanese quince juice is approximately twice that of lemon juice, ranging 229.2–459.0 mg/100 mL, compared to 84.8–196.8 mg/100 mL in lemon juice (*8*, *11*).

Juice concentration is a common industrial practice that facilitates storage and transportation (*12*). Widely used juice concentrates include apple, lemon, orange, grapefruit, tangerine, lime, pomegranate, pineapple, apricot and mango. The quality of juice concentrates is influenced by several factors, including the physiological maturity of the fruit, the quality of the raw material, processing technology and storage conditions (*11*). One key quality indicator is the content of 5-hydroxymethylfurfural (HMF), an organic compound formed by the dehydration of reducing sugars, mainly hexoses, under acidic conditions (*13*).

Pectin, a high-molecular-mass polysaccharide present in plant cell walls, plays a crucial role in juice quality. It consists of negatively charged functional groups distributed along a backbone of d-galacturonic acid monomers linked *via* α-1→4-glycosidic bonds. Pectin can hinder juice clarification by interacting with other compounds in the matrix (*14*). To address this, enzymatic treatments using hydrolytic enzymes such as pectinases and amylases are commonly employed to break down pectin and polysaccharides (*14*). Pectinases hydrolyze pectin, disrupt pectin-protein complexes and reduce viscosity, thereby improving filtration efficiency and lowering energy costs.

Enzymes, as efficient biocatalysts, are widely used at various stages of juice production. Specifically, pectinases play a vital role in improving clarity and stability by reducing viscosity (*15*). Industrial depectinization processes typically use commercial enzyme mixtures containing pectinases, pectinesterases, polygalacturonases, cellulases, and pectin lyases (*16*).

Despite the increasing interest in Japanese quince juice, only one preliminary study has investigated the preparation and evaluated the quality of Japanese quince juice concentrate (*17*). The study found that treating juice with enzyme preparation Ultrazym 100 G, which primarily has polygalacturonase activity, resulted in improved quality indices. Given the high juice yield, dominance of organic acids and antioxidant properties, Japanese quince juice and its concentrate could serve as viable alternatives to lemon juice, potentially enhancing food preservation and extending shelf life.

With the increasing demand for innovative and health-promoting food products, Japanese quince juice has the potential to become a niche product with global market appeal (*18*). The limited availability of research articles analysing Japanese quince juice as a potential acidifying alternative to lemon juice prompted the design of this study. This research aims to determine the effects of different hydrolytic enzymes and a clarifying agent on the quality indicators of the juice. Specifically, the study seeks to assess how these treatments influence parameters such as clarity, pectin content and overall juice quality, thereby identifying optimal methods for processing the juice into a concentrate that could serve as an effective acidifying alternative to lemon juice. It is hypothesized that the application of hydrolytic enzymes and a clarifying agent will significantly improve juice quality by enhancing clarity, reducing pectin content and preserving or boosting its bioactive properties. Furthermore, it is proposed that processing the juice into a concentrate using these treatments will yield a product that can serve as an effective acidifying alternative to lemon juice, offering comparable or superior bioactive benefits while maintaining high product quality.

## MATERIALS AND METHODS

The following materials were used: ethanol 96.3 % (Kalsnava Distillery, Madona, Latvia), Folin-Ciocalteu phenol reagent, 2,2-diphenyl-1-picrylhydrazyl (DPPH˙) reagent, potassium chloride, potassium hexacyanidoferrate(II), anhydrous sodium carbonate, iron trichloride hexahydrate (Sigma-Aldrich Chemie GmbH, Merck, Darmsadt, Germany), 2,4,6-tripyridyl-s-triazine, tannic acid, ascorbic acid (Fluka, Dublin, Ireland), aluminium chloride hexahydrate (Alfa Aesar, Thermo Fisher Scientific, Munich, Germany), sodium nitrite (VWR, Val-de-Marne, France), sodium acetate trihydrate (AppliChem Chemicals, Darmstadt, Germany), (+)-catechin, gallic acid monohydrate, sodium hydroxide pellets, hydrochloric acid (Merck, Rahway, NJ, USA) and activated carbon (Eiro Plus, Kyiv, Ukraine). Enzymes EnartisZym 1000 S, EnartisZym RS and EnartisZym EZ Filter, and clarifying agent Neoclar AF were purchased from Enartis, Trecate, Italy. Analytical standard l-ascorbic acid, as well as acetonitrile (MeCN) and formic acid (HCOOH) of liquid chromatography-mass spectrometry (LC-MS) grade were purchased from Merck KGaA (Darmstadt, Germany). Ultrapure water was produced using reverse osmosis PureLab Flex Elga water purification system (Veolia Water Technologies, Paris, France).

The research used whole, fully ripe Japanese quince (*Chaenomeles japonica* L.) fruits, harvested in late August at the Institute of Horticulture in Dobele, Latvia (location: 56°36'39.9"N 23°17'48.8"E). The ripeness of the fruit was determined using a protocol (ME 05 CHE 2017) developed at the Institute of Horticulture (LatHort), Ceriņi, Latvia, based on the evaluation of fruit surface and seed colour. This assessment was conducted organoleptically, using the following scale: 1 represents unripe fruit, characterized by green skin and white seeds, 2–4 represent intermediate ripeness stages, and 5 indicates fully ripe fruit, with yellow skin and dark brown seeds, where the colour corresponds to botanical colour standards. After harvesting, fruits (50 kg) were washed, dried, packed in airtight and moisture-proof polypropylene bags, frozen and stored at (−18±1) °C for 3 months until processing. The fruits were thawed at room temperature for 24 h, the juice was obtained with a Basket press 60K (Voran Maschinen GmbH, Pichl bei Wels, Austria). Immediately after squeezing, the Japanese quince juice was divided into five portions of 3 L each for enzymatic treatment with two replications. Raw juice served as the control sample.

### Japanese quince juice clarification with enzyme preparations

A total of three clarification enzymes and a clarifying agent Neoclar AF were used in the study. Table 1 shows the characteristic parameters and amounts of enzymes and clarifying agent added to 1 L of juice.

**Table 1:** Properties of the enzymes and clarifying agent used for the treatment of Japanese quince juice

Enzyme (abbreviation)	Component	*w*/%	*t*(treatment)/h	Temperature/°C	Amount of added substance
EnartisZym 1000 S (1000 S)	Polygalacturonase	12.5–15	2	50±0.5	0.02 g
EnartisZym RS (RS)	1,3(4)-β-endo-glucanase	5–7	2	50±0.5	0.03 mL
	Pectin lyase	1–3			
	Pectinesterase	0.5–1			
	Polygalacturonase	0.5–1			
EnartisZym EZ Filter (EZ)	1,3(4)-β-endo-glucanase	5–7	2	50±0.5	0.04 mL
	Pectinesterase	5–7			
	Pectin lyase	3–5			
	Polygalacturonase	3–5			
Neoclar AF (Bentonite)	Bentonite powder	70–80	48	4±1	1.5 g
	Activated carbon	5–7			
	Gelatine powder	not indicated			

The conditions for the enzymatic treatment of the juice were selected based on the manufacturer’s instructions and considering the observations made by other researchers (*19*). Enzymatic treatment of juice samples was performed using a water bath (WB-4MS; Biosan, Riga, Latvia). The samples were subjected to thermal treatment at 90 °C for 3 min to terminate the catalytic activity of enzymes. Then the processed juice samples were cooled and filtered through Whatman filter no. 1. Instead of enzymatic treatment, one juice sample was clarified using the clarifying agent Neoclar AF under refrigeration conditions at 4 °C for 48 h, The juice was then filtered through Whatman filter No. 1. All the filtrates were collected for further analysis and preparation of the concentrate.

### Preparation of juice concentrate

For concentration, the filtered Japanese quince juice was evaporated in wide, low glass beakers using an open-form evaporation method in a laboratory water bath (JP Selecta™ Precisdig; JP Selecta, Barcelona, Spain). The evaporation was controlled by estimating the soluble solids content, which was done periodically with a refractometer (PAL-1; Atago, Tokyo, Japan) until the concentrate reached (50±1) % Brix. The obtained juice concentrate was cooled, filled in closed containers and stored at 4 °C until further analysis.

### Physicochemical analyses

The following chemical indices were determined for Japanese quince juice and its concentrate: soluble solids, titratable acidy, pH, clarity, the content of total vitamin C, proanthocyanidins, phenolics, pectins and antioxidant activity by free radical scavenging activity DPPH˙ and Fe(III) reducing antioxidant power (FRAP) assays.

#### Soluble solids content

Soluble solids content (% Brix) in juice and concentrate was determined using a digital refractometer type Pal-1 (Atago).

#### Clarity

Clarity was determined by measuring the absorbance at 660 nm using a spectrophotometer UV 1800 (Shimadzu, Tokyo, Japan). Deionized water was used as a reference according to Sin *et al.* (*20*).

#### Titratable acidity

Titratable acidity (TA) of samples was assessed using 10 mL water extracts, which were titrated with 0.1 mol/L NaOH to a pH=8.1 according to general guidelines for objective tests (*21*). TA was expressed as percentage of citric acid equivalent in fresh mass (FM).

#### Total phenolic content

The total phenolic content (TPC) in the juice and its concentrate was determined by a photometric method using the Folin-Ciocalteu reagent, as described by Singleton *et al.* (*22*). The sample was prepared, in three replications, as follows: 1 mL of juice or (0.50±0.01) g of the concentrate were transferred to a 50-mL flask. Then, 30 mL of 80 % ethanol was added, and the mixture was vortexed and extracted in an ultrasonic bath (Sonorex Digitec DT 512 H; BANDELIN electronic GmbH&Co. KG, Berlin, Germany) for 30 min, centrifuged and filtered. The TPC in the sample was expressed as mg or g gallic acid equivalent (GAE) per 100 g FM. For the evaluation of antioxidant activity (DPPH˙ and FRAP), the same extracts were used.

#### Total proanthocyanidin content

The total proanthocyanidin content was estimated according to the method of Price and Butler, as described by Paaver *et al.* (*23*). To prepare the samples, 1 g of juice or 0.5 g of concentrate was transferred to a 100-mL flask, to which 50 mL of water were added and boiled for 30 min. After filtration through a Whatman filter, the solution was transferred to a 100-mL flask and water was added to reach 100 mL mark. Aliquots of 0.5 mL were transferred to vials, 1 mL of 1 % K_3_Fe(CN)_6_ and 1 mL of 1 % FeCl_3_ were added, and final volume was adjusted to 10 mL with water. After 5 min, the solutions were measured spectrophotometrically at 720 nm. The results were expressed as mg or g of catechin equivalent (CE) per 100 g FM.

#### Pectin content

Pectin was determined by the carbazole method (*24*). Pectin was isolated from the juice and its concentrate by leaching with 96.0 % ethanol, and from the residues by extraction with diluted 1 M NaOH solution. After adding 0.1 % carbazole solution and concentrated sulfuric acid to the extract, the sample was heated at 85 °C for 15 min. The determination was based on the reaction of pectin with carbazole, in an excess of concentrated sulfuric acid, for the development of a pink colour. The intensity of the colour was measured with a spectrophotometer UV 1800 (Shimadzu) at 525 nm. The amount of pectin was expressed as mg of galacturonic acid equivalent (GalAE) per 100 mL of juice or g of GalAE per 100 g of concentrate FM.

#### Vitamin C content

Vitamin C was determined by high performance liquid chromatography (HPLC) according to EN 14130:2003 standard (*25*) and expressed as mg per 100 g FM.

#### DPPH˙ free radical scavenging activity

The DPPH˙ free radical scavenging activity was determined using the 2,2-diphenyl-1-picrylhydrazyl assay according to Floegel *et al*. (*26*), with minor modifications. Briefly, the extract (0.1 mL) was reacted with 2.9 mL of DPPH˙ solution (0.0039 g DPPH˙ in 100 mL of 96.0 % ethanol). Absorbance of the sample was measured at 515 nm using a spectrophotometer (UV-1800; Shimadzu Corporation). The radical scavenging activity of the sample was expressed in mmol of Trolox equivalents (TE) per 100 g FM.

#### Fe(II) reducing antioxidant power

The FRAP was determined according to Benzie and Strain (*27*) with some modifications. The FRAP reagent solution was prepared from 300 mmol acetate buffer, pH=3.6, 10 mol 2,4,6-tripyridyl-s-triazine (TPTZ) solution in 40 mmol of HCl, and 20 mmol of FeCl_3_·6H_2_O solution. The fresh working solution was prepared by mixing 25 mL of acetate buffer, 2.5 mL of TPTZ solution and 2.5 mL of FeCl_3_·6H_2_O solution. Sample extracts (0.150 mL) reacted with 2.850 mL of FRAP solution for 10 min in the dark. The changes in absorbance from red to blue were determined at 593 nm. The absorbance results were converted using a calibration curve of the standard and expressed as mmol of TE per 100 g of juice and mol of TE per 100 g of concentrate FM.

#### 5-Hydroxymethylfurfural content

The chromatography analysis for the determination of 5-hydrosymethylfurfural (HMF) was carried out following the method reported by Gökmen and Acar (*28*) using a Shimadzu LC-40 Nexera system (Shimadzu Corporation). The system comprised a column oven CTO-40C, autosampler SIL-40CX3, solvent delivery module LC-40D X3, degassing unit DGU-405 and system controller SCL-40.

Separation was performed on an aqueous C18 column (250 mm×4.6 mm, 5 µm; PerkinElmer, Ltd., Waltham, MA, USA) under isocratic elution mode at a column temperature of 35 °C. The mobile phase consisted of acetonitrile and water in a 10:90 ratio, with a sample injection volume of 10 µL. The total analysis time was up to 8 min, with a flow rate of 1.0 mL/min, and detection was carried out at a wavelength of 280 nm using an SPD-M40 photodiode array detector (Shimadzu Corporation). The concentration of 5-HMF was expressed in µg per 100 g of the sample.

### Statistical analysis

Experimental data were processed using one-way analysis of variance (ANOVA) and IBM^®^ SPSS^®^ Statistics v. 23 (*29*). The mean arithmetic value and standard deviation were calculated for the obtained results using MS Office Excel 2019 v. 18008 (build 10416.20073) (Microsoft Corporation, Redmond, WA, USA). Tukey’s multiple range test was used to specify significant differences (p<0.05) among the studied samples. Analytical hierarchy process (AHP) was used to evaluate the clarification efficiency of enzyme treatment. The key quality characteristics of the samples of Japanese quince juice concentrate were carefully selected for evaluation, with clarity identified as the primary parameter. Given that the focus of the study was the development of Japanese quince juice concentrate as an alternative to lemon-based acidifiers, acidity was identified as the second most critical quality attribute. An index ranging from 1 to 9 was assigned to the evaluation indicators of the quince concentrate samples according to their relative importance: clarity 9, titratable acidity 7, pectin 5, antioxidant activity (DPPH˙) 3, and vitamin C 1. The priority coordinate vectors were calculated following the AHP method recommended in the literature (*30*).

## RESULTS AND DISCUSSION

### Japanese quince juice

The effect of enzymatic treatment on the quality indicators of Japanese quince juice was examined in this study, with the results summarized in Table 2. The results show that treatment with enzymes significantly (p<0.05) improved the clarity of the juice, achieving almost 90 % transparency compared with the control (untreated) sample. The clarity values of clarified juice ranged from 0.021 to 0.053. The most pronounced effect of enzymes on the clarity of the juice was observed in the following order: 1000 S (91.2 %)>bentonite (88.3 %)>EZ (86.2 %)>RS (77.9 %). Hmid *et al*. (*31*) found that pomegranate juice clarity increases as the concentration of the used enzymes (particularly pectinase and protease) increases. The authors noted that applying enzymes at volume fractions from 0.05 to 0.5 % substantially improved juice clarity. The potential applicability of polygalacturonase in the food industry was demonstrated by Amobonye *et al*. (*32*), who observed that enzymatic treatment of pear juice with polygalacturonase substantially improved its clarity, positively affecting the reduction of the browning index and decreasing turbidity.

**Table 2:** Chemical composition, antioxidant activity and clarity of Japanese quince juice after treatment with enzymes and clarifying agent

Parameter	Sample				
	Control	1000 S	RS	EZ	Bentonite
TA/%	(2.35±0.06)^ab^	(2.30±0.00)^a^	(2.35±0.06)^ab^	(2.30±0.00)^a^	(2.45±0.06)^b^
*w*(soluble solid)/% Brix	(7.8±0.1)^a^	(7.8±0.2)^a^	(7.65±0.06)^a^	(7.8±0.1)^a^	(7.65±0.06)^a^
Clarity (*A*_660 nm_)	(0.24±0.01)^c^	(0.02±0.00)^a^	(0.05±0.00)^b^	(0.03±0.00)^a^	(0.03±0.00)^a^
*w*(vitamin C)/(mg/100 g)	(72.8±0.6)^b^	(69.0±1.0)^a^	(68.4±0.4)^a^	(69.4±0.9)^a^	(69.1±0.8)^a^
*w*(total phenolics as GAE)/(mg/100 g)	(326.2±2.9)^b^	(319.4±3.6)^ab^	(320.4±2.1)^ab^	(316.2±5.7)^a^	(316.6±3.1)^a^
*w*(total proanthocyanidins as CE)/(mg/100 g)	(314.3±5.2)^d^	(287.4±4.8)^c^	(281.2±2.7)^c^	(258.2±2.7)^b^	(242.4±2.1)^a^
DPPH˙ scavenging as *n*(TE)/(mmol/100 g)	(2.60±0.02)^a^	(2.70±0.01)^b^	(2.75±0.01)^b^	(2.70±0.06)^b^	(2.75±0.06)^b^
FRAP as *n*(TE)/(mmol/100 g)	(93.0±5.3)^b^	(87.0±1.6)^a^	(91.8±3.8)^b^	(87.20±1.2)^a^	(87.6±2.8)^a^
pH	(2.83±0.00)^d^	(2.78±0.00)^b^	(2.71±0.00)^a^	(2.82±0.00)^c^	(2.83±0.00)^d^

Values are mean±S.D. (*N*=4). Values with different letters in superscript in the same row are significantly different (p<0.05).

TA=titratable acidity, GAE=gallic acid equivalent, CE=catechin equivalent, TE=Trolox equivalent, 1000 S=EnartisZym 1000 S, RS=EnartisZym RS, EZ=EnartisZym EZ Filter. DPPH and FRAP values are expressed on fresh mass basis

The titratable acidity and soluble solids content in the control Japanese quince juice sample, which was frozen and thawed before analysis, were 2.35 and 7.80 % Brix, respectively, while the pH was 2.83. Similar results were reported by Nowak *et al*. (*33*), who found an almost identical composition of fresh (unprocessed) juices, with a pH=2.8 of quince juice and a soluble solids content of 7.5 % Brix. Enzymatic treatment of Japanese quince juice with pectolytic enzymes and bentonite affected titratable acidity (p=0.002) and pH (p=0.001). In contrast, the soluble solids content did not change significantly (p>0.05) compared with the control sample. A more significant difference was determined with the pectinase treatment of pomegranate juice, where the initial pH (4.13) was reduced from 3.69 (0.5 % pectinase) to 3.82 (0.05 % pectinase) during clarification (*31*). Amobonye *et al*. (*32*) reported a similar pH reduction after clarification of pear juice with polygalacturonase, where the difference between the control and treated juice was 3.6 %. The reduction in pH during juice clarification is believed to result from the release of galacturonic acid, which is a pectin degradation product.

**Fig. 1:**
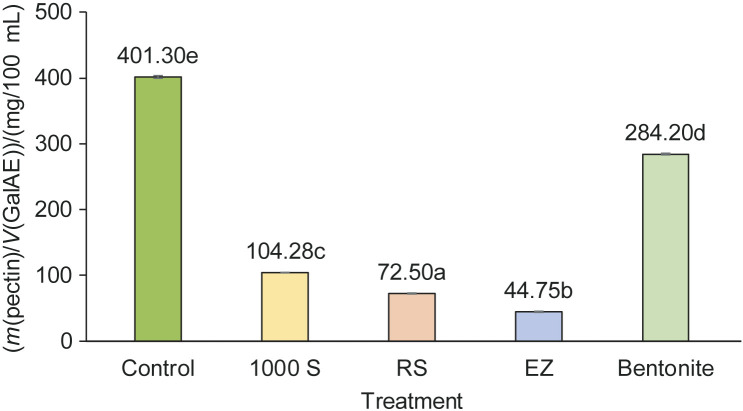
The content of pectins in Japanese quince juice after the treatment with enzymes and Neoclar AF (bentonite). Values are mean±S.D. (*N*=3). Bars with different letters show significantly different values (p<0.05). 1000 S=EnartisZym 1000 S, RS=EnartisZym RS, EZ=EnartisZym EZ Filter, GalAE=galacturonic acid equivalent

Treatment of Japanese quince juice with hydrolytic enzymes and bentonite significantly reduced the pectin content in the samples (p<0.05) compared to the control, which was an important objective of the study (Fig. 1). The highest depectinization efficiency was observed after enzymatic treatment with enzyme EZ and filtration, as nearly 88.9 % of the pectin present in the control sample was removed. A significantly lower but relevant reduction in pectin content was observed in the juice treated with RS and 1000 S, with a pectin content decrease of 81.9 and 74.0 %, respectively, compared to the initial value. Treatment with bentonite resulted in a significantly lower depectinization efficiency, reducing pectin content by 29.2 % compared to the initial amount. This can be attributed to the composition of bentonite and the mechanism that removes pectin from liquids. According to the datasheet for the Neoclar AF preparation, bentonite does not contain pectin-degrading enzymes; the primary mechanism of pectin removal is the interaction of negatively charged bentonite and activated charcoal with positively charged high-molecular-mass compounds, particularly proteins, leading to their precipitation. Since the formation of protein-pectin complex, which occurs more intensively at low pH, greatly affects the clarity of the juice (*34*), partial clarification can be achieved by adding bentonite to quince juice. A similar observation was reported by Valdhuber and Pulko (*35*), who highlighted the ability of bentonite with a small addition of sodium to clarify apple juice effectively.

The total phenolic content (TPC) in Japanese quince juice, expressed as GAE on fresh mass basis, ranged from 316.55 to 326.25 mg/100 g, with the control juice sample containing the highest content and the bentonite- and enzyme-treated EZ sample the lowest. However, no significant influence of juice clarification on TPC was observed, since the reduction amounted to 1.8–3.1 % compared to the control sample. The results of this study do not coincide with the findings reported by Martino *et al.* (*36*) and Candrawinata *et al.* (*37*), which indicate either an increase or decrease in TPC after juice clarification.

The amount of vitamin C on fresh mass basis in Japanese quince juice samples ranged from 68.35 to 72.8 mg/100 g, with the control sample containing the highest mass fraction and the enzyme-treated samples, *i.e*. 1000 S and RS, the lowest. It was observed that the addition of enzymes to the juice for clarification negatively affected the vitamin C content. After clarification, the amount of vitamin C decreased by 4.6–6.1 % compared to the control sample. The decrease in vitamin C content is perhaps due to the sensitivity of this vitamin to heat, light and oxygen, to which the samples were exposed during juice clarification. However, research by Radziejewska-Kubzdela (*38*), which focused on the analysis of bioactive compounds in strawberry depending on the type of treatment, *i.e*. ultrasonic, thermal and enzymatic, did not reveal a direct influence of enzymatic treatment with pectin-degrading enzymes on vitamin C content.

The mass fraction of proanthocyanidins on fresh mass basis in Japanese quince juice, expressed as CE, ranged from 242.4 to 314.3 mg/100 g, with the control juice sample containing the highest mass fraction and the samples treated with EZ and bentonite the lowest. Similar to vitamin C, the clarification process negatively affected the content of this biocompound with the percentage reduction from 8.5 to 22.9 %. The 1000 S enzyme had the most negligible effect, while bentonite had the greatest. Studies on tannin adsorption in water treatment (*39*) have shown a strong interaction between various substrates and tannins, including activated carbon, clay and bentonite. This observation is supported by the findings of Youn *et al.* (*40*), who reported that activated carbon or bentonite achieved tannin mitigation efficiencies of 26.9 and 23.1 %, respectively, in apple juice. A study on the treatment of apple juice with various clarifiers conducted by Mazrou *et al.* (*41*) and Oszmiański *et al.* (*42*) indicated that bentonite and gelatine (even with the addition of chitosan) adsorb and reduce the content of phenolic compounds, including proanthocyanidins.

When analyzing the antiradical activity of Japanese quince juice, expressed as TE on fresh mass basis, it was observed that the control sample had a value of 2.6 mmol/100 g, while samples subjected to clarification showed slightly higher radical scavenging activity, increased by 3.8 to 5.8 %, with the samples treated with RS and bentonite having the highest values and 1000 S and EZ the lowest. The results of the FRAP assay showed significantly higher values, expressed as TE on fresh mass basis, ranging from 87.00 to 92.95 mmol/100 g, with the control quince juice sample having the highest value, and 1000 S and EZ the lowest. According to FRAP analysis, a percentage reduction in antioxidant activity was observed, with the loss ranging from 1.3 to 6.4 %. Enzymes 1000 S and EZ, and bentonite had the most substantial negative impact, while RS had negligible effects. A similar observation was made by Mazrou *et al.* (*41*), who reported that after treatment with bentonite, the turbidity of grape juice decreased and it became more transparent; however, the TPC (including proanthocyanidins) was substantially decreased. This finding is supported by data provided by Dey and Banerjee (*43*), which show that the clarification of apple juice using enzymes and activated charcoal resulted in a 19.8 % reduction in TPC compared to cloudy juice samples. Similarly, activated carbon has been shown to modify the volatile composition of fruits, including grape juice (*44*), potentially affecting both the flavour and colour of juice and wine. In a study by Liu *et al*. (*45*), activated carbon treatment was found to reduce ester concentrations in stored apples, which play a key role in fruit aroma. The action of hydrolytic enzymes, in turn, is aimed at breaking the ester bonds between macromolecules and active substances, such as phenolic compounds (*46*) and volatile organic compounds (*47*). This process facilitates the release of bound fractions, which are typically involved in various biochemical reactions or contribute to the bioactivity of the substances. Essentially, hydrolysis by these enzymes cleaves the ester linkages, resulting in the release of components that were previously bound within larger structures (*48*). However, Amobonye *et al.* (*32*) indicated that treatment of pear juice with the pectin-degrading enzyme polygalacturonase preserved antioxidant potential and TPC, as no significant changes were detected after treatment. Chemical compounds in different juice samples may react differently when treated with complex enzymes or activated carbon, which generally affects antioxidant activity and volatile compound composition.

### Japanese quince juice concentrate

The concentration of fruit juices requires the partial removal of water without changing the solid composition, retaining all original solid components, such as fruit carbohydrates, minerals and vitamins, in the concentrated solution, which is essential for the food industry (*12*). The quality indicators of Japanese quince juice concentrate as a final product determine its suitability for the food industry, which, according to the purpose of the research, would be classified as acidifier, similar to lemon juice concentrate. Based on the quality indicators of lemon juice concentrate available on the market, Japanese quince juice samples were steamed to a soluble dry matter content of (50.5±0.5) % Brix after enzymatic treatment and filtration. The chemical composition, antioxidant activity and physical properties of the juice concentrate are summarized in Table 3.

**Table 3:** Chemical composition, antioxidant activity and clarity of Japanese quince juice concentrate after treatment with enzymes and clarifying agent

Parameter	Sample				
	Control	1000 S	RS	EZ	Bentonite
TA/%	(29.78±0.00)^a^	(29.8±0.3)^a^	(30.17±0.00)^a^	(29.94±0.00)^a^	(30.0±0.2)^a^
*w*(soluble solid)/% Brix	(50.4±0.1)^a^	(50.2±0.2)^a^	(50.5±0.1)^a^	(50.3±0.2)^a^	(50.40±0.09)^a^
Clarity (*A*_660 nm_)	(1.16±0.00)^e^	(0.33±0.00)^a^	(0.42±0.00)^c^	(0.48±0.00)^d^	(0.40±0.00)^b^
*w*(vitamin C)/(mg/100 g)	(245.0±0.6)^c^	(235.1±2.2)^ab^	(231.4±1.4)^a^	(236.1±1.7)^b^	(236.9±0.9)^b^
*w*(total phenolics as GAE)/(g/100 g)	(3.34±0.05)^b^	(3.1±0.1)^a^	(3.2±0.1)^ab^	(3.13±0.08)^ab^	(3.2±0.1)^ab^
*w*(total proanthocyanidins as CE)/(g/100 g)	(2.93±0.01)^e^	(2.32±0.01)^d^	(2.29±0.01)^c^	(1.90±0.01)^b^	(1.75±0.01)^a^
DPPH˙ scavenging as *n*(TE)/(mmol/100 g)	(6.1±0.3)^a^	(5.4±0.4)^a^	(5.7±0.2)^a^	(5.5±0.8)^a^	(5.5±0.4)^a^
FRAP as *n*(TE)/(mmol/100 g)	(0.55±0.02)^a^	(0.51±0.04)^a^	(0.51±0.01)^a^	(0.51±0.00)^a^	(0.51±0.00)^a^
pH	(2.55±0.01)^b^	(2.56±0.01)^b^	(2.53±0.01)^a^	(2.56±0.01)^b^	(2.56±0.01)^b^

Values are mean±S.D(*N*=4). Values with different letters in superscript in the same row are significantly different (p<0.05).

TA=titratable acidity, GAE=gallic acid equivalent, CE=catechin equivalent, TE=Trolox equivalent, FM=fresh mass, 1000 S=EnartisZym 1000 S, RS=EnartisZym RS, EZ=EnartisZym EZ Filter. DPPH and FRAP values are expressed on fresh mass basis

Titratable acidity and pH did not differ significantly between juice samples (p>0.05), showing values from 29.78 to 30.17 % and 2.53 to 2.56, respectively. According to Tamer *et al.* (*49*), lemon juice concentrate (Limkon Fruit Juice Concentrate Facilities, Adana, Turkey) with 45.4 % soluble solids content had a titratable acidity of 15.66 g/100 g, which is almost half that observed in Japanese quince juice concentrate samples.

The results showed that treatment with enzymes considerably (p<0.05) improved the clarity of Japanese quince juice concentrate, with an average transparency of 64.9 % compared with the control (untreated) sample. The clarity values of clarified and concentrated juice ranged from 0.33 to 0.48, compared to 1.16 in the control juice sample. The most pronounced effects of the juice pretreatment on the juice concentrate clarity can be arranged in the following order: 1000 S (71.6 %)>bentonite (65.5 %)>RS (63.8 %)>ES (58.6 %). The use of enzymatic treatment with enzyme 1000 S, which contains polygalacturonase as the sole pectin-degrading enzyme, produced a satisfactory juice concentrate clarity and can be considered by the industry for potential use in fruit juice clarification.

**Fig. 2:**
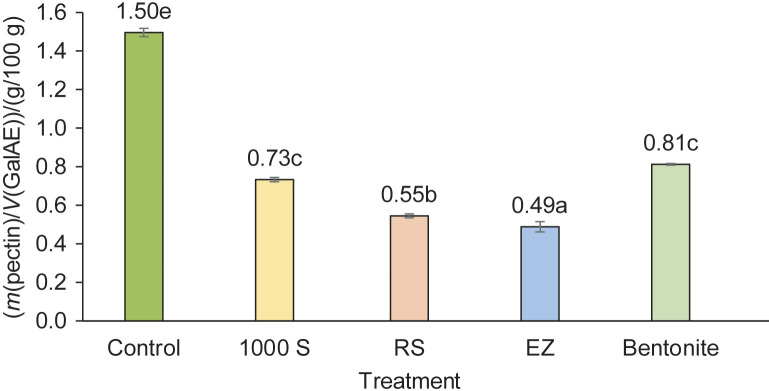
The content of pectins in Japanese quince juice concentrates after the treatment with enzymes and Neoclar AF (bentonite). Values are mean±S.D. (*N*=3). Bars with different letters show significantly different values (p<0.05). 1000 S=EnartisZym 1000 S, RS=EnartisZym RS, EZ=EnartisZym EZ Filter, GalAE=galacturonic acid equivalent

The pectin content, expressed as GalAE on fresh mass basis, was 1.50 g/100 g in the control juice concentrate sample. In the samples treated with enzymes, it ranged from 0.49 to 0.73 g/100 g, and in the bentonite-treated sample, it was 0.81 g/100 g (Fig. 2). The most significant reduction in pectin from juice concentrate was achieved when enzymes with cellulolytic and pectinolytic activities in the RS and EZ preparations were used. The reduction of pectin content in these samples was 63.3 and 67.3 %, respectively. While a significantly (p<0.05) lower but relevant decrease in the pectin content was reached using 1000 S and bentonite, the loss of pectin was 51.3 and 46.0 %, respectively. According to these data, a combination of two or three enzyme preparations with both cellulolytic and pectinolytic activity, *i.e.* RS or EZ and 1000 S, can deliver sufficient quality of fruit juice in terms of clarity.

The amount of vitamin C on fresh mass basis in Japanese quince juice concentrate samples ranged from 231.4 to 245.0 mg/100 g, with the control juice sample containing the highest amount and the enzyme-treated samples, *i.e.* RS and 1000 S, the lowest. Burdurlu *et al.* (*50*) reported a very similar value of vitamin C, 225.0 mg/100 g, in lemon juice concentrate. It was observed that the addition of enzymes to the juice for clarification negatively affected the vitamin C content. After clarification, the amount of vitamin C in the concentrate, as in the juice, decreased by 3.3–5.5 % compared to the control sample. As shown, the mass fraction of vitamin C in the juice concentrate increased on average 3.4 times compared to the juice, indicating that the evaporation conditions used to remove excess water from the juice may affect the content of bioactive compounds. In a study by Hellín *et al.* (*17*), the vitamin C content in Japanese quince juice concentrate with a soluble solids content of 70 % Brix, achieved using a laboratory-scale evaporator at 40 °C and 4 kPa vacuum, increased from 96 to 1048 mg/100 mL, corresponding to an increase of more than 10 times.

The TPC in Japanese quince juice concentrate, expressed as GAE on fresh mass basis, ranged from 3.07 to 3.34 g/100 g, with the control juice concentrate having the highest content and the enzyme-treated sample 1000 S the lowest. The observed values were substantially higher than those reported by Tamer *et al.* (*49*), 116 g/100 g, for lemon juice concentrate. As a result of concentration, the TPC in Japanese quince juice concentrate increased almost 10 times. The most significant decrease in the TPC was observed in the juice concentrate treated with 1000 S, followed by EZ.

The mass fraction of proanthocyanidins in Japanese quince juice concentrate, expressed as CE on fresh mass basis, ranged from 1.75 to 2.93 g/100 g. The control juice had the highest mass fraction, while the samples treated with bentonite and EZ had the lowest. Overall, the manipulations to which the juice samples were subjected during clarification, namely enzymatic or bentonite treatment, filtration and evaporation, resulted in a 20.8 to 40.4 % loss of proanthocyanidin content. Similar to the juice, the most favourable effect of processing on the content of proanthocyanidins was observed in the juice concentrate obtained after treatment of juice with enzyme preparations 1000 S and RS.

The use of cellulolytic and pectolytic enzymes and bentonite did not significantly (p>0.05) affect the antioxidant activity of Japanese quince juice concentrate according to DPPH˙ and FRAP assays. DPPH˙ radical scavenging activity values, expressed as TE on fresh mass basis, ranged from 5.4 to 6.1 mmol/100 g, while FRAP values, also expressed as TE on fresh mass basis, ranged from 0.51 to 0.55 mol/100 g. The control sample showed slightly higher values in both antioxidant activity assays than the juice subjected to the clarification process. The observed DPPH˙ values differ from those reported by Tamer *et al*. (*49*) for lemon juice concentrate, which were 12.1 mmol/100 g, and are on average 5.5 times lower than those observed in Japanese quince juice concentrate. Substantial differences were also observed between the FRAP values obtained in this study and those reported by Tamer *et al.* (*49*) for lemon juice concentrate, indicating that Japanese quince juice concentrate had a much stronger reducing power than lemon concentrate. Among the four fruit juice concentrates evaluated, tangerine, grape, lemon and lime, Oikeh *et al.* (*51*) identified the grape juice as the superior radical scavenger; the value was 36.42 mmol/g of extract, which is nearly seven times higher than observed in the present study. The reported differences are due primarily to the nature of the samples, the content of biologically active compounds and, to a lesser extent, fruit processing conditions and sample preparation methods.

As previously reported, 5-hydroxymethylfurfural (HMF) in the sample may indicate partial or complete dehydration of sugars, mainly hexoses, under acidic conditions or if the sample was exposed to heat treatment. HMF is widely used as a marker of quality deterioration, thermal processing and other adulteration practices. Based on this, the HPLC analysis was conducted to determine the amount of HMF in the prepared Japanese quince juice concentrate after heat and time exposure. The HPLC analysis showed no presence of HMF in the prepared juice concentrate, indicating that the selected conditions for developing the juice concentrate were appropriate. However, the presence of HMF in boiled juice samples (*52*) and in lemon juice and its concentrate (*53*) confirmed that thermal exposure was the main cause. Moreover, during 120 days of storage, the HMF content in lemon juice concentrate nearly doubled, reaching 503.20 μg/L. Burdurlu *et al.* (*50*) reported that HMF accumulation in citrus juice concentrates increased with increasing storage temperature, and eight weeks of storage at 45 °C was equivalent to about 2.7 times longer storage at 37 °C. Higher storage temperatures also negatively affected the vitamin C content of citrus fruit concentrate, which decreased more rapidly during storage. The importance of controlling HMF during storage is still relevant and must be considered at the product development stage.

**Fig. 3:**
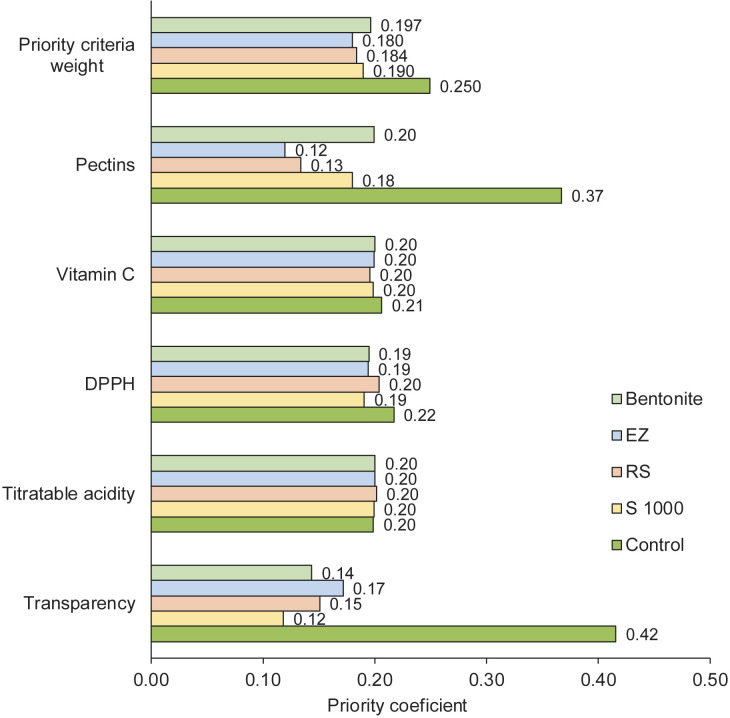
The results of the analytical hierarchy process analysis. 1000 S=EnartisZym 1000 S, RS=EnartisZym RS, EZ=EnartisZym EZ Filter

The analytical hierarchy process (AHP) was used to evaluate the effect of enzymatic treatment on the quality characteristics of Japanese quince juice concentrate. Importance vectors were calculated by AHP, considering the clarity of the concentrate (after juice clarification) and the main indicators characterizing the chemical content: titratable acidity and pectin content, antioxidant activity and vitamin C content. According to AHP, the lowest priority coefficient value is the most important criterion. The calculated vectors were as follows: clarity=0.04, titratable acidity=0.18, pectin=0.21, DPPH˙=0.25 and vitamin C=0.32. The results of the AHP are summarized in Fig. 3. Evaluating the quality indicators of the juice concentrate according to the AHP, it can be concluded that the treatment of juice with the pectin-degrading enzyme 1000 S positively affected the clarity of the concentrate (priority coefficient 0.12). Similarly, the enzymatic treatment of juice samples with the 1000 S preparation led to increased DPPH˙ radical scavenging activity, as indicated by the corresponding priority coefficient of 0.19. The most apparent effect of 1000 S is observed when compared to the untreated control sample. The AHP analysis highlights the importance of RS and EZ enzymatic preparations in effectively removing pectic substances from the juice, with priority coefficients of 0.13 and 0.12, respectively. According to the AHP summary, considering all selected priority criteria, the EnartisZym enzyme preparations EZ and RS, both declared to have cellulolytic and pectinolytic activities, achieved the lowest priority coefficient values of 0.18. The presence of cellulose-degrading enzymes along with pectinases resulted in the most efficient removal of high-molecular-mass compounds from the colloidal systems of the juice and can be considered suitable for juice clarification. However, in light of these results, further studies are needed to understand the effect of cellulolytic enzymes on juice clarification. Process optimization and operational conditions during fruit juice clarification can deliver the desired quality of the final product with relative stability of nutrients.

## CONCLUSIONS

This study elucidated the impact of three pectin-degrading enzymes and a clarifying agent containing a blend of bentonite and activated charcoal on the quality characteristics of Japanese quince (*Chaenomeles japonica* L.) juice and its concentrate. The results showed that all applied clarifying preparations can be considered suitable by the industry to improve the appearance of fruit juice and reduce antinutrients such as proanthocyanidins. Data obtained using the analytical hierarchy process (AHP) identified the superiority of the enzymatic preparation EnartisZym 1000 S, which contains polygalacturonase as the sole pectin-degrading enzyme. However, considering overall scores from the AHP analysis, the enzyme EnartisZym EZ Filter, which has both cellulolytic and pectolytic activities, was found to be the most efficient, ensuring quality characteristics of Japanese quince juice concentrate equivalent to those of commercially available products such as lemon juice concentrate. Sequential clarification processing, namely enzymatic treatment, filtration, and concentration or evaporation of Japanese quince juice resulted in the following values of the final product: titratable acidity 29.94 %, pH=2.56 and vitamin C mass fraction 236.1 mg/100 g. Overall, the developed product can be considered an alternative to lemon juice concentrates that offers not only preservative and acidifying properties, but also nutritional benefits to the products to which it is added. Further research should focus on an in-depth study of Japanese quince juice concentrate, including its use in the preparation of various products.
